# Significance of Inverse Planning With Variable Dose Rate and the Segment Shape Optimization in Dynamic Conformal Arcs Using the High-Definition Dynamic Radiosurgery Platform for Single Brain Metastases

**DOI:** 10.7759/cureus.89500

**Published:** 2025-08-06

**Authors:** Kazuhiro Ohtakara, Kojiro Suzuki

**Affiliations:** 1 Department of Radiation Oncology, Kainan Hospital Aichi Prefectural Welfare Federation of Agricultural Cooperatives, Yatomi, JPN; 2 Department of Radiology, Aichi Medical University, Nagakute, JPN

**Keywords:** brain metastases, dose conformity, dose gradient, dynamic conformal arcs, dynamic conformal arc therapy, inverse planning, segment shape optimization, stereotactic radiosurgery, variable dose rate, x-ray voxel monte carlo

## Abstract

Purpose

This planning study aimed to clarify the significance of inverse planning with variable dose rate (VDR) and the segment shape optimization (SSO) in the quality and efficiency of dynamic conformal arcs (DCA) using the high-definition dynamic radiosurgery (HDRS) platform for stereotactic radiosurgery (SRS) of single brain metastases (BMs).

Materials and methods

Twenty clinical BMs were included, with the gross tumor volume (GTV) ranging from 0.33 cc to 48.09 cc (median: 7.05 cc). The HDRS platform included the 5-mm leaf-width, 160-leaf collimator Agility® (Elekta AB, Stockholm, Sweden) and the Monaco® planning system (Elekta AB). Prior to the main comparison, the high-precision leaf positions (HPLP) values of between five and 20 in the SSO were compared to determine which was optimal in six lesions. Using the constant dose rate (CDR) optimization as a baseline (the CDR group), the effects of changing to VDR (the VDR group), and further adding the SSO with the suitable HPLP value (the SSO group) on the DCA planning were investigated. The same prescription dose was assigned to the GTV *D*_V-0.01 cc_ (minimum dose of GTV minus 0.01 cc).

Results

The HPLP value of 20 in the SSO (SSO_20) was suitable in terms of the total calculation time (tCT), the total monitor units (MU) per fraction, and the GTV dose conformity and gradients. The tCT was significantly longer in the order of the SSO_20, the VDR, and the CDR. The total MU was the highest in the SSO_20, and the MU assignments to the three arcs were automatically optimized in each group. The change from CDR to VDR significantly improved the GTV dose conformity, the appropriateness of dose attenuation margin outside the GTV, the steepness of dose gradient outside the GTV, and the concentric lamellarity of dose increase 2-4 mm inside the GTV boundary. The addition of the SSO_20 further significantly improved the GTV dose conformity, the dose attenuation margin, the steepness, and the concentric lamellarity of dose gradients outside and inside the GTV boundary. In the SSO_20, the beam segments were shaped by anisotropic leaf adaptations to the GTV boundary with extensions of some of the leaf edges beyond the GTV boundary (minus leaf margins) and the practically <5 mm variable widths of the outermost leaves in the leaf movement direction through the dynamic shielding by the diaphragms (jaws), along with the position controls of both the leaves and the diaphragms in 0.1 mm increments.

Conclusions

The inverse planning with VDR and the SSO_20 significantly improved the quality of DCA plans in terms of the dose conformity and gradients outside and inside the GTV boundary. However, the SSO_20 with VDR required longer tCT and higher total MU per fraction. The SSO_20 with VDR was recommended for DCA-based SRS planning using the HDRS for BMs.

## Introduction

Stereotactic radiosurgery (SRS) is an essential therapeutic avenue, especially for symptomatic brain metastases (BMs) to shrink the lesions quickly and sufficiently and thereby to improve the relevant symptoms sustainably [1-3]. The proper implementation of SRS using a general-purpose linac allows for rapid delivery of SRS at nearby facilities and seamless coordination with systemic therapy. Dynamic conformal arcs (DCA) with forward planning have traditionally been the commonly used irradiation method for SRS with a multileaf collimator (MLC) [4]. In the DCA, the arc arrangement, the collimator angles, each beam weight (i.e., monitor units (MU) per arc), and the leaf margins to a target volume (TV) are determined by the intentions and/or discretion of planners [4,5]. In particular, the leaf margins need to be set appropriately depending on the intended TV dose inhomogeneity (e.g., 70% isodose covering) and the leaf characteristics [4-8]. However, fixed leaf margin settings or specific % coverage of the TV are problematic in ensuring consistency of dose prescription to the TV boundary [4,5,7].

The Elements® treatment planning system (TPS) (Brainlab AG, Munich, Germany) provides efficient DCA with inverse planning, enabling simultaneous irradiation of multiple targets with a single isocenter [9-11]. In the inverse planning, the leaf margin for each TV is automatically adjusted to be an isotropic leaf margin that may even be a negative value, depending on the planner’s intention [9-11]. The irradiation fields are simply and dynamically created by shaping conformal beam apertures to the target contour or blocking them completely as the arc rotates [9-11]. However, the more irregular the shape of the lesion and/or the closer the distance between multiple lesions, the more likely it is that dose conformity will deteriorate in DCA with either the forward or inverse planning [12]. To improve dose distribution in the complex cases, a dummy structure with a modified three-dimensional shape of the TV may be employed as the modified TV (mTV) [12-14]. However, in order to attain an appropriate dose distribution, it is necessary to repeatedly modify the mTV shape and recalculate, making the treatment plan quite complicated and time-consuming [12].

The high-definition dynamic radiosurgery (HDRS) platform, including the 5-mm leaf-width, 160-leaf MLC Agility® (Elekta AB, Stockholm, Sweden) and the Monaco® TPS (Elekta AB), also supports SRS planning and the implementation [15-17]. The HDRS is a major component of a general-purpose linac capable of high precision irradiation, highlighting the constituents for providing image-guided frameless SRS [15,17]. The HDRS also enables DCA with the following features: inverse planning with the optimization algorithm similar to volumetric-modulated arcs (VMA), variable gantry rotation speed with constant dose rate (CDR), and highly accurate dose calculation with the X-ray voxel Monte Carlo (XVMC) algorithm [18-20]. Furthermore, variable dose rate (VDR) and the segment shape optimization (SSO), both of which are essential for optimizing volumetric modulated arcs (VMA), can be selected as options for the DCA planning [18]. However, the dosimetric characteristics and the clinical utility of the DCA planning using the HDRS, in particular the significance of the SSO with VDR, have not been fully elucidated.

This planning study was therefore conducted to clarify the significance of the SSO with VDR in the inverse planning for DCA-based SRS with the HDRS for single BMs. Specifically, using the DCA plan with CDR as a reference, we examine the effects of changing to VDR and then adding the SSO on the quality and efficiency of the DCA planning.

## Materials and methods

This study was performed as part of a study (approval number: 20240830-01) approved by the Clinical Research Review Board of Kainan Hospital, Aichi Prefectural Welfare Federation of Agricultural Cooperatives.

Twenty lesions were extracted from 19 cases for which multi-fraction SRS was performed previously in our hospital, and each lesion was treated as a single metastasis. The gross tumor volume (GTV) ranged from 0.33 cc to 48.09 cc (median value: 7.05 cc; interquartile range (IQR): 2.08, 26.34 cc).

The treatment platform consisted of the Agility® MLC (Elekta AB), mounted in a linac Infinity® (Elekta AB) with a flattening filter-free 6 MV X-ray beam, and the TPS Monaco® (version 5.51.10; Elekta AB) [15,21]. The irradiation isocenter was set at the center of each GTV, to which three arcs with a rotation range of 120º were arranged for each GTV to minimize the beam path lengths [22]. The arc rotations were arranged so as not to extend beyond the horizontal plane of the isocenter to the caudal side. The three arcs uniformly consisted of one coplanar arc with the collimator angle of 0° and two non-coplanar arcs (NCAs) with the collimator angles of 45° and 90° [22]. The couch of two NCAs' couches was rotated 60° clockwise and counterclockwise, respectively, so that the orbits of the three arcs trisected the cephalad hemisphere. The increment (Inc) mechanical limiting parameter for each arc was set to 20°.

For each GTV, three different DCA plans were created with all the optimization settings identical, except for the sequencing parameters, as described in Tables 1-2.

**Table 1:** The unified settings, except for the sequencing parameters, for plan optimization of dynamic conformal arcs (DCA). *Although the term IMRT is included, these are merely parameters for optimizing DCA. **The minimum volume is set according to the coverage value of the *D*_V-0.01 cc_, minimum dose of target volume (TV) minus 0.01 cc, (*D*_>95%_) for each gross tumor volume (GTV). Rx: prescription; IMRT: intensity-modulated radiotherapy; RMS: root mean square; Min.: minimum

Item	Setting details		
Prescription	Rx dose (Gy): 43.000		
	Number of fractions: 5		
IMRT Constraints (Pareto)	Structure	Cost function	Parameter settings
	GTV	Target penalty	Prescription (Gy): 43.000
			Minimum volume (%): 97.00-99.98**
	Patient (body contour)	Conformality	Relative isoconstraint: 0.01
			Margin around target: 8 cm
			Multicriterial +
		Quadratic Overdose	Maximum dose (Gy): 43.000
			RMS dose excess (Gy): 0.020
			Multicriterial +
			Shrink structures (cm): GTV 0.20
IMRT Prescription Parameters*	Beamlet width (cm): 0.20		
	Target margin: very tight (0-1 mm)		
	Avoidance margin: very tight (0-1 mm)		
Calculation Properties	Grid spacing (cm): 0.10		
	Calculation dose deposition to: medium		
	Statistical uncertainty (%): 1.00 per calculation		

**Table 2:** Comparison of the four different settings of the sequencing parameters for DCA. DCA: dynamic conformal arcs; CDR: constant dose rate; VDR: variable dose rate; SSO: segment shape optimization; NA: not available

Optimization Group	Constant Dose Rate	Segment Shape Optimization	High-Precision Leaf Positions
CDR	+	-	NA
VDR	-	-	NA
SSO_5	-	+	5 (default)
SSO_20	-	+	20 (maximum)

In the general dose prescription method for the GTV *D*_98%_, which covers a certain percentage of the GTV, the larger the GTV, the larger the amount of uncovered GTV, which is more likely to result in impaired local control [23]. Therefore, the same prescription dose of 43.000 Gy in five fractions was uniformly assigned to the GTV *D*_V-0.01 cc_, the minimum dose to the GTV minus 0.01 cc [23]. After the completion of optimization, each GTV coverage with the prescription dose (43.000 Gy) was rescaled according to each coverage value (97.00-99.98%) of the GTV *D*_V-0.01 cc_ [23]. A change in the GTV dose after rescaling was recorded to the third decimal place as the rescaling ratio as described previously [24]. The tCT was recorded from the optimization console [24].

The parameters used to compare the dose distributions are shown in Table 3.

**Table 3:** Evaluation metrics adopted for the comparison of the dose distributions. *This metric can also be interpreted as follows: the higher the value, the steeper the dose increase inside the GTV boundary, although the higher value ​​may occur due to over-coverage of the GTV by the prescription isodose. GTV: gross tumor volume; *D*_V-0.01 cc_: minimum dose to a target volume (TV) minus 0.01 cc (*D*_>95%_); IDS: isodose surface; *D*_0.01 cc_: minimum dose to 0.01 cc, receiving the near maximum dose, of TV (*D*_<5%_); PIV: prescription isodose volume; IIDV: irradiated isodose volume; *D*_eIIV_: minimum dose to the IIDV equivalent to a TV

Evaluation Items	Metrics	Definitions	Interpretation
GTV dose inhomogeneity	GTV *D*_V-0.01 cc_ % IDS (%)	GTV *D*_V-0.01 cc_ (%) relative to *D*_0.01 cc_ (100%)	The lower the value, the more heterogeneous
GTV dose conformity	PIV spillage (cc)	IIDV of GTV *D*_V-0.01 cc_ minus GTV	The smaller the value, the better the conformity
	GTV *D*_eIIV_ (%)	minimum dose (%) to IIDV equivalent to GTV, relative to GTV *D*_V-0.01 cc_ (100%)	The closer to 100%, the better the conformity*
	GTV *D*_eIIV_ coverage (%)	GTV coverage (%) by *D*_eIIV_	The higher the value, the better the conformity
Steepness of dose decrease at 2 mm outside GTV boundary	GTV + 2 mm *D*_eIIV_ (%)	minimum dose (%) to IIDV equivalent to GTV + 2 mm, relative to GTV *D*_V-0.01 cc_ (100%)	The lower the value, the steeper the dose decrease (gradient) outside the GTV boundary
Concentric lamellarity of the dose gradient at 2 mm outside GTV boundary	GTV + 2 mm *D*_eIIV_ coverage (%)	GTV + 2 mm coverage (%) by *D*_eIIV_	The higher the value, the better the concentric layering of the dose gradient outside the GTV boundary
Steepness of dose gradient outside GTV	75% or 50% PIV spillage (cc)	IIDV of 75% or 50% of GTV *D*_V-0.01 cc_ minus GTV	The smaller the value, the steeper the dose gradient outside the GTV boundary
Steepness of dose increase at 2-4 mm inside the GTV boundary	GTV – 2 or 4 mm *D*_eIIV_ (%)	minimum dose (%) to IIDV equivalent to GTV – 2 or 4 mm, relative to GTV *D*_V-0.01 cc_ (100%)	The higher the value, the steeper the dose increase inside the GTV boundary
Concentric lamellarity of dose gradient at 2-4 mm inside GTV boundary	GTV – 2 or 4 mm *D*_eIIV_ coverage (%)	GTV – 2 or 4 mm coverage (%) by *D*_eIIV_	The higher the value, the better the concentric layering of the dose gradient inside the GTV boundary

Six lesions were extracted, and the treatment plans were compared to determine whether the high-precision leaf positions value of five or 20 was optimal, and the more optimal value was then adopted for comparison of the 20 lesions. The GTV-2 mm and GTV-4 mm were created only for GTVs of ≥1.26 cc (17 lesions) and ≥1.71 cc (16 lesions), respectively, to ensure the minimum meaningful volumes for evaluation [24].

The statistical analyses were based on paired nonparametric tests, using the BellCurve for Excel® (version 4.05; Social Survey Research Information Co., Ltd., Tokyo, Japan). The distributions of numerical variables were shown as box-and-whisker plots (BWPs). In the BWP, the whiskers indicate the nearest values ≤1.5 times the IQR. The cross marks beyond the lines denote the outliers >1.5 times the IQR. Two numerical variables were compared using the Wilcoxon signed-rank test (WSRT). The correlation between two numerical variables was evaluated using Spearman’s rank correlation coefficient (SRCC). Statistical significance was considered at p<0.05 and expressed on a three-level scale: p<0.05 (*), p<0.01 (**), and p<0.001 (***). Significant p-values were marked in blue in the figures.

## Results

**Figure 1:**
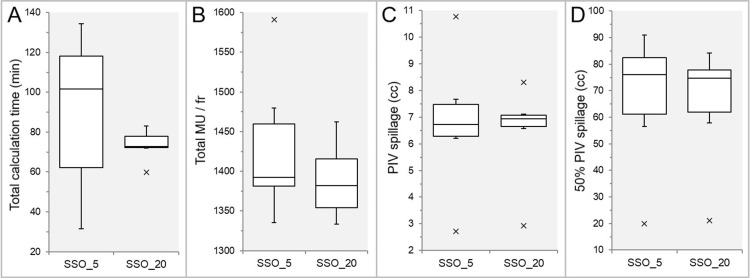
Comparisons of the segment shape optimization with the high-precision leaf positions value of between five and 20. The images show box-and-whisker plots (BWPs) (A-D) for comparisons of the total calculation time (tCT) (A), the total monitor units (MU) per fraction (fr) (B), the prescription isodose volume (PIV) spillage outside the gross tumor volume (GTV) (C), and the 50% PIV spillage volume (D) in the six lesions. SSO_5: segment shape optimization (SSO) with the high-precision leaf positions value of five (default); SSO_20: SSO with the high-precision leaf positions value of 20 (highest).

The main results comparing five and 20 of the high-precision leaf positions value (SSO_5 vs. SSO_20) are shown in Figure 1.

In the SSO_20, the tCT was shorter with little variability (Figure 1A); the total MU per fraction was lower (Figure 1B); and the 50% prescription isodose volume (PIV) spillage was smaller (Figure 1D), compared to the SSO_5. In addition, the third quartile to the maximum value of the PIV spillage was smaller, while the median value was higher (Figure 1C). In the 20 lesions, comparisons were made between the CDR and VDR and then the VDR and SSO_20.

**Figure 2:**
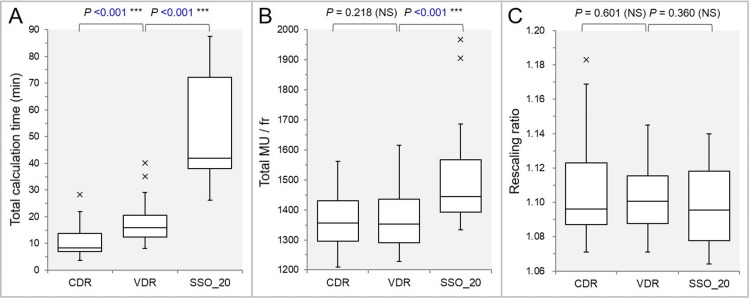
Comparisons of the optimization time, the total MU per fraction, and the rescaling ratio. The images show BWPs (A-C), along with the results of the Wilcoxon signed-rank test (WSRT), for comparisons of the tCT (A); the total MU per fraction (fr) (B); and the rescaling ratio (C). MU: monitor units; CDR: constant dose rate; VDR: variable dose rate; SSO: segment shape optimization; SSO_20: SSO with high-precision leaf positions value of 20; NS: not significant; BWPs: box-and-whisker plots: tCT: total calculation time

The tCT was significantly longer in the order of the SSO_20, the VDR, and the CDR (Figure 2A).

The total MU per fraction was significantly higher in the SSO_20 than in the VDR, while there was no significant difference between the VDR and the CDR (Figure 2B). There were no significant differences in the rescaling ratios between the SSO_20 and the VDR and between the VDR and the CDR (Figure 2C).

**Figure 3:**
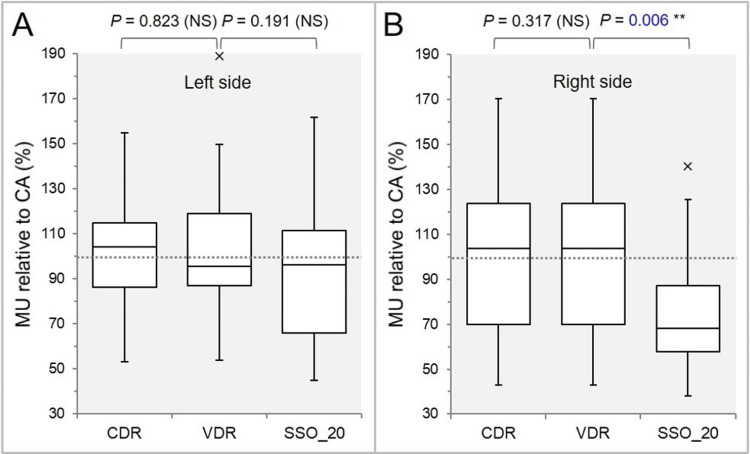
Comparisons of the MU value of each non-coplanar arc relative to that of the coplanar arc. The images show BWPs (A,B), along with the results of WSRT, for comparisons of the MU (%) of two non-coplanar arcs (A: left side, B: right side), relative to those of the coplanar arcs (CA, 100%). The dotted lines in A and B indicate 100%, which is the same as the MU value of the CA. MU: monitor units; CDR: constant dose rate; VDR: variable dose rate; SSO: segment shape optimization; SSO_20: SSO with high-precision leaf positions value of 20; NS: not significant; BWPs: box-and-whisker plots; WSRT: Wilcoxon signed-rank test

The MU for each NCA relative to those of the coplanar arc (100%) was significantly lower in the right-sided NCA of the SSO_20 than in the CDR (Figure 3B), while there was no significant difference in the left-sided one (Figure 3A).

**Figure 4:**
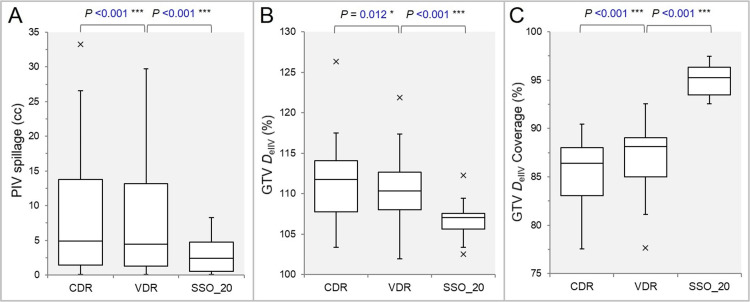
Comparisons of the GTV dose conformity. The images show BWPs (A-C), along with the results of WSRT, for comparisons of the PIV spillage volume outside the GTV (A); the GTV *D*_eIIV_ (%) relative to the *D*_V-0.01 cc_ (100%) (B); and the GTV coverage value by the *D*_eIIV_ (C). GTV: gross tumor volume; CDR: constant dose rate; VDR: variable dose rate; SSO: segment shape optimization; SSO_20: SSO with high-precision leaf positions value of 20; PIV: prescription isodose volume; *D*_eIIV_: minimum dose to the irradiated isodose volume equivalent to target volume (TV); BWPs: box-and-whisker plots; WSRT: Wilcoxon signed-rank test; *D*_V-0.01 cc_: minimum dose to TV minus 0.01 cc

The PIV spillage volume was significantly smaller in the SSO_20, the VDR, and the CDR in that order (Figure 4A).

The GTV *D*_eIIV_, the minimum dose to the irradiated isodose volume equivalent to the reference target volume, was significantly lower in the order of the SSO_20, the VDR, and the CDR (Figure 4B), and the GTV coverage by the *D*_eIIV_ was significantly higher in the order of the SSO_20, the VDR, and the CDR (Figure 4C).

**Figure 5:**
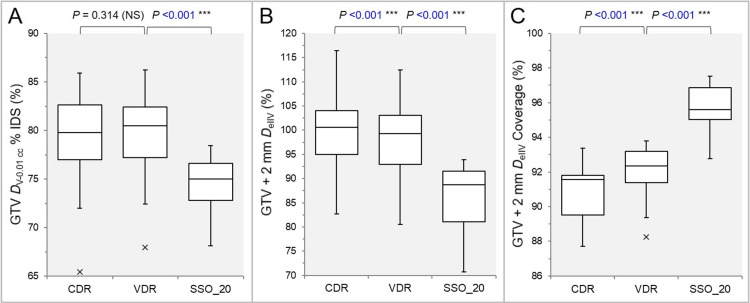
Comparisons of the GTV dose inhomogeneity and the appropriateness of dose attenuation margin. The images show BWPs (A-C), along with the results of WSRT, for comparisons of the GTV *D*_V-0.01 cc_ (%) relative to the *D*_0.01 cc_ (100%) (A); the GTV + 2 mm *D*_eIIV_ (%) relative to the GTV *D*_V-0.01 cc_ (100%) (B); and the coverage value of GTV + 2 mm by the *D*_eIIV_ (C). GTV: gross tumor volume; CDR: constant dose rate; VDR: variable dose rate; SSO: segment shape optimization; SSO_20: SSO with high-precision leaf positions value of 20; *D*_V-0.01 cc_: minimum dose to target volume (TV) minus 0.01 cc; IDS: isodose surface; *D*_eIIV_: minimum dose to the irradiated isodose volume equivalent to TV; BWPs: box-and-whisker plots; WSRT: Wilcoxon signed-rank test; *D*_0.01 cc_: minimum dose to 0.01 cc of TV

The GTV *D*_V-0.01 cc_ (%) relative to the *D*_0.01 cc_ (100%) was significantly lower in the SSO_20 than the VDR, while there was no significant difference between the VDR and the CDR (Figure 5A).

The *D*_eIIV_ of GTV + 2 mm was significantly lower in the order of the SSO_20, the VDR, and the CDR (Figure 5B). The lowest was 70.7% of the GTV of 0.33 cc in the SSO_20. The GTV + 2 mm coverage by the *D*_eIIV_ was significantly higher in the order of the SSO_20, the VDR, and the CDR (Figure 5C). In the SSO_20, the GTV + 2 mm *D*_eIIV_ significantly increased with increasing GTV (rho = 0.932, p<0.001 ***). In addition, the GTV + 2 mm coverage value by the *D*_eIIV_ significantly increased as the GTV increased (rho = 0.869, p<0.001 ***).

**Figure 6:**
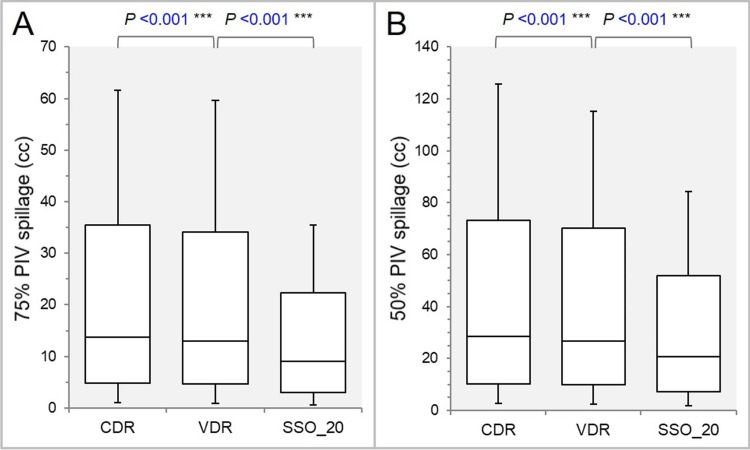
Comparisons of the steepness of dose gradient outside the GTV. The images show BWPs (A,B), along with the results of WSRT, for comparisons of the 75% (A) and 50% (B) PIV spillage volumes outside the GTV. GTV: gross tumor volume; CDR: constant dose rate; VDR: variable dose rate; SSO: segment shape optimization; SSO_20: SSO with high-precision leaf positions value of 20; PIV: prescription isodose volume; BWPs: box-and-whisker plots; WSRT: Wilcoxon signed-rank test

The 75% and 50% PIV spillage volumes were significantly smaller in the order of the SSO_20, the VDR, and the CDR (Figures 6A-6B).

**Figure 7:**
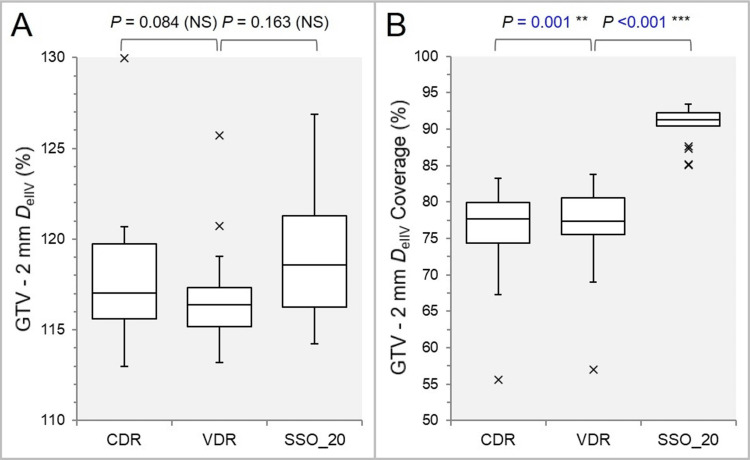
Comparisons of the steepness of dose increase and the concentric lamellarity at 2 mm inside the GTV boundary. The images show BWPs (A,B), along with the results of WSRT, for comparisons of the GTV-2 mm *D*_eIIV_ (%) relative to the GTV *D*_V-0.01 cc_ (100%) (A); and the coverage value of GTV-2 mm by the *D*_eIIV_ (B). GTV: gross tumor volume; CDR: constant dose rate; VDR: variable dose rate; SSO: segment shape optimization; SSO_20: SSO with high-precision leaf positions value of 20; *D*_eIIV_: minimum dose to the irradiated isodose volume equivalent to target volume (TV); NS: not significant; BWPs: box-and-whisker plots; WSRT: Wilcoxon signed-rank test; *D*_V-0.01 cc_: minimum dose to TV minus 0.01 cc

There were no significant differences in the *D*_eIIV_ of the GTV-2 mm between the three groups (Figure 7A), while the GTV-2 mm coverage value by the *D*_eIIV_ was significantly higher in the order of the SSO_20, the VDR, and the CDR (Figure 7B).

**Figure 8:**
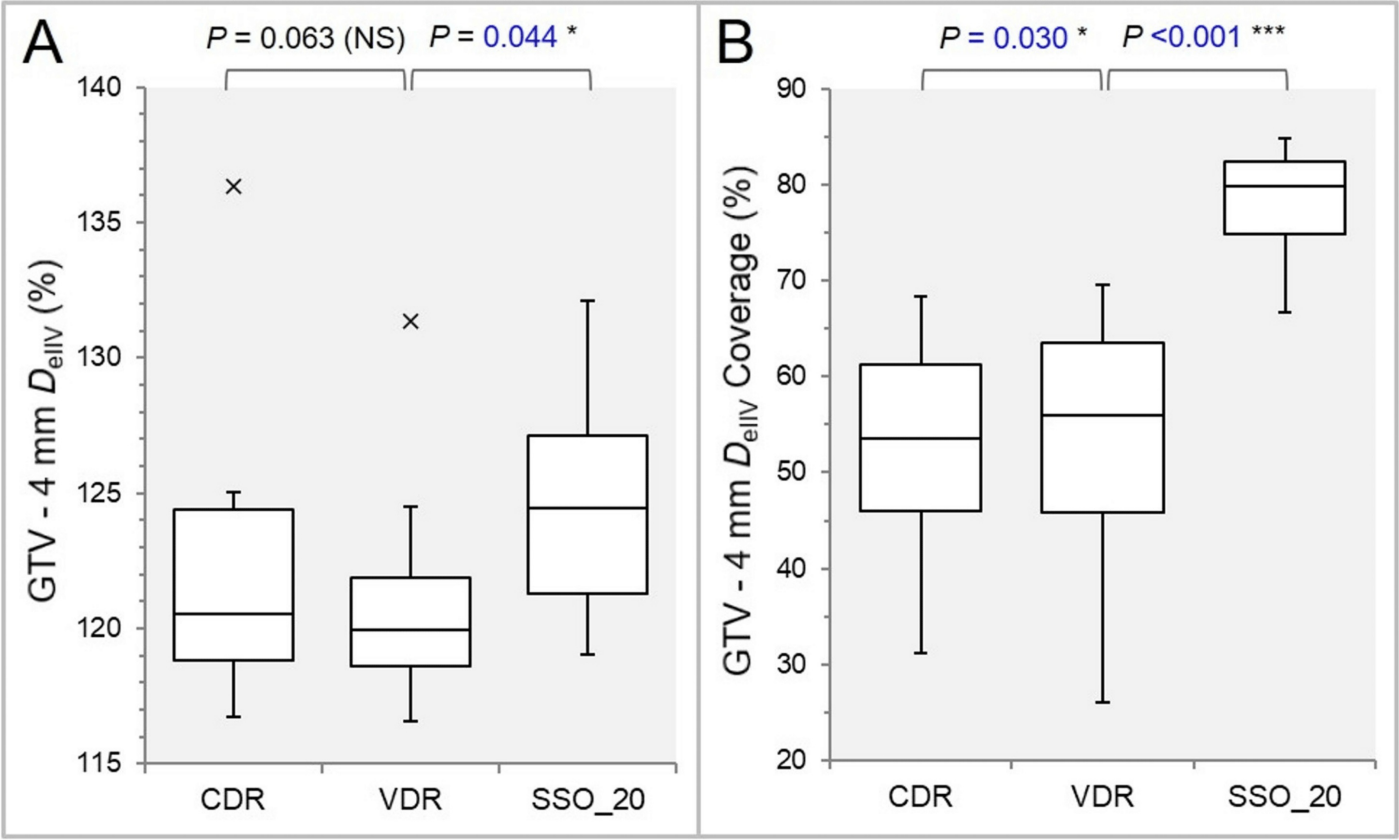
Comparisons of the steepness of dose increase and the concentric lamellarity at 4 mm inside the GTV boundary. The images show BWPs (A,B), along with the results of WSRT, for comparisons of the GTV-4 mm *D*_eIIV_ (%) relative to the GTV *D*_V-0.01 cc_ (100%) (A); and the coverage value of GTV-4 mm by the *D*_eIIV_ (B). GTV: gross tumor volume; CDR: constant dose rate; VDR: variable dose rate; SSO: segment shape optimization; SSO_20: SSO with high-precision leaf positions value of 20; *D*_eIIV_: minimum dose to the irradiated isodose volume equivalent to target volume (TV); NS: not significant; BWPs: box-and-whisker plots; WSRT: Wilcoxon signed-rank test; *D*_V-0.01 cc_: minimum dose to TV minus 0.01 cc

The *D*_eIIV_ of the GTV-4 mm was significantly higher in the SSO_20 than the VDR, while there was no significant difference between the VDR and the CDR (Figure 8A).

**Figure 9:**
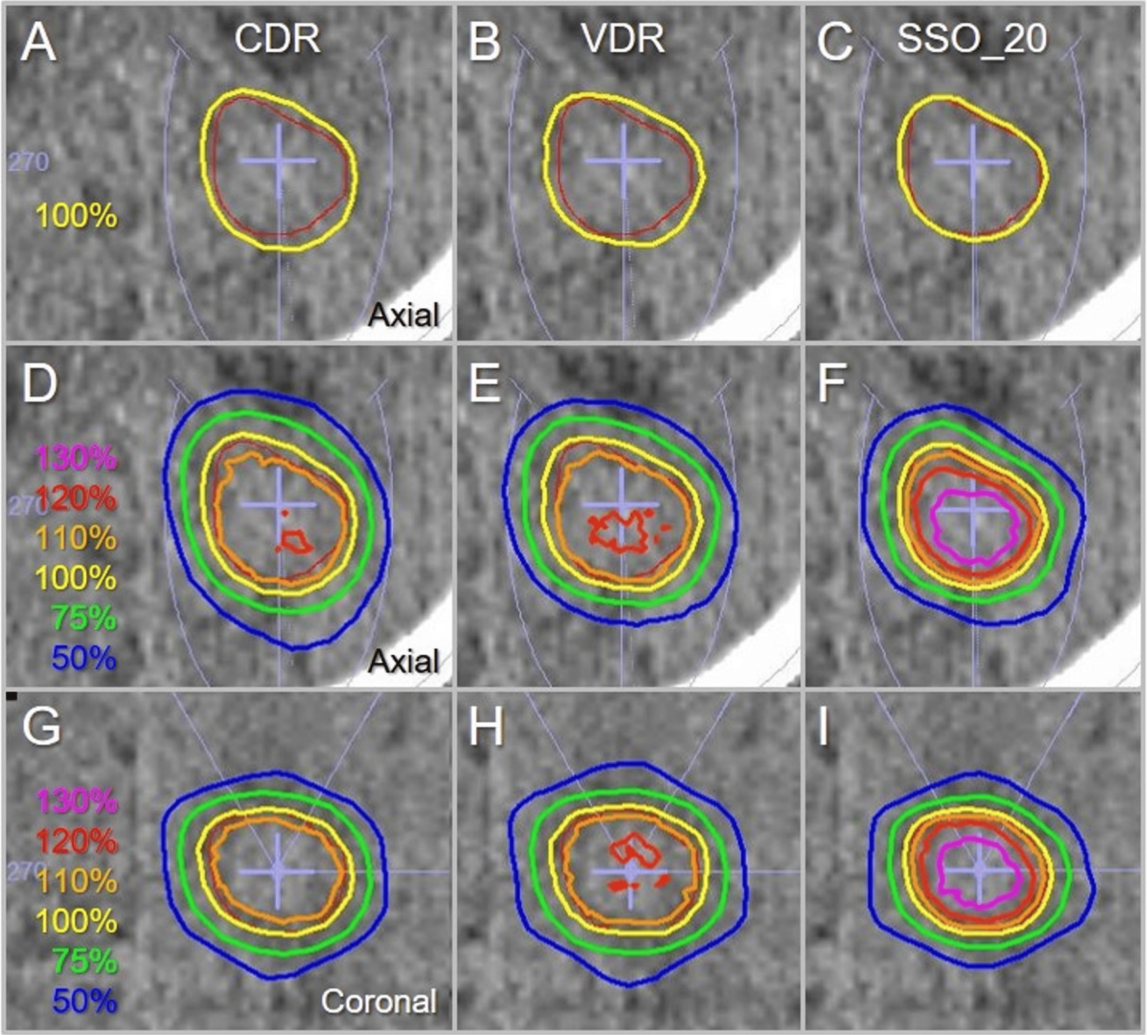
Comparison of the dose distributions for GTV of 3.20 cc. Computed tomographic images, axial (A-F); and coronal views (G-I), of a patient with a BM in the left occipital lobe (A-I) are shown. The GTV outline (red), arc arrangements (light purple), and representative isodoses in the CDR (A, D, G), VDR (B, E, H), and SSO_20 (C, F, I) are superimposed. The isodose lines are shown as the relative values to the GTV *D*_V-0.01 cc_ (100%, yellow). GTV: gross tumor volume; CDR: constant dose rate; VDR: variable dose rate; SSO_20: segment shape optimization with high-precision leaf positions value of 20; BM: brain metastasis; *D*_V-0.01 cc_: minimum dose to target volume minus 0.01 cc

The GTV-4 mm coverage value by the *D*_eIIV_ was significantly higher in the order of the SSO_20, the VDR, and the CDR (Figure 8B). The representative dose distributions for the GTV of 3.20 cc are shown in Figure 9.

The dose distribution in the SSO_20 was the best in terms of prescription isodose conformity to the GTV boundary and the steepness and concentric lamellarity of dose gradients inside and outside the GTV boundary.

**Figure 10:**
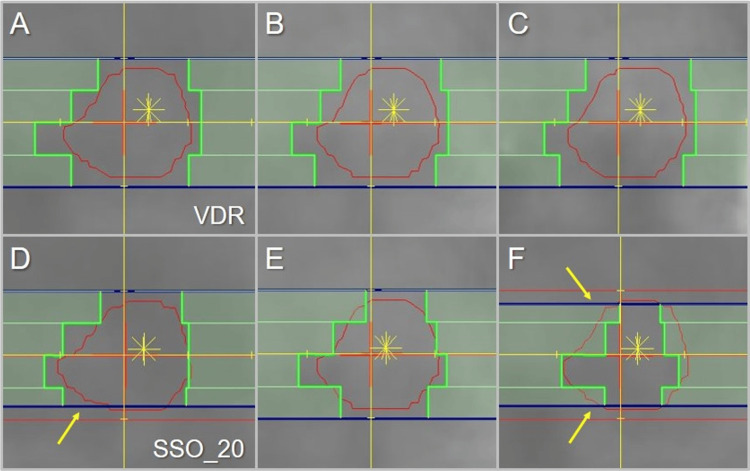
Comparison of the beam’s eye views of the coplanar arcs for the GTV of 3.20 cc. The images show the beam’s eye views (A-F) of the representative three segments of the coplanar arcs for the VDR (A-C) and the SSO_20 (D-F). Arrows (D, F) indicate the <5 mm variable widths of some outer leaves by the dynamic involvement of diaphragms (dark blue), which is effective only with the SSO_20. The asterisks denote each highest dose area. VDR: variable dose rate; SSO_20: segment shape optimization with a high-precision leaf positions value of 20

The representative beam segments of the coplanar arcs for the DVR and the SSO_20 are shown in Figure 10.

Each leaf edge maintains a roughly constant margin to the GTV boundary in the VDR, while some of the edges extend quite far inside the GTV boundary in the SSO_20 (i.e., minus leaf margins). In addition, some of the outer leaves in the SSO_20 essentially function as <5 mm variable widths by the dynamic involvement of diaphragms (jaws) or the more in-depth jaw tracking.

The beam statistics of the SSO_20 plan are shown in Table 4.

**Table 4:** The beam statistics of the SSO_20 plan for the GTV of 3.20 cc. *The MU% of the second (left-sided) and third (right-sided) non-coplanar arcs is the relative ratio of the MU% of the first (left-sided) coplanar arc to 100%. SSO_20: segment shape optimization with a high-precision leaf positions value of 20; MU: monitor unit; Fx: fraction; Min.: minimum; CP: control point; Max.: maximum

Arc (sequence)	1	2	3
Couch	0.0°	300.0°	60.0°
Collimator Angle	0.0°	45.0°	90.0°
Number of Segments	27	21	18
Total MU / Fx (%)*	774.39 (100.0%)	418.12 (54.0%)	370.82 (47.9%)
Min. CP MU / Fx	3.29	3.29	3.29
Max. CP MU / Fx	83.33	55.40	91.08

The number of segments per arc and the allocation of MU values to each arc varied substantially. The difference in dose rate was up to 27.7 times.

## Discussion

The inverse planning with changing from CDR to VDR and adding the SSO_20 significantly improved the quality of dose distributions in the DCA using the HDRS. However, the SSO_20 with VDR had the longest tCT and the highest total MU per fraction, leading to the poorest efficiency of treatment planning and delivery. The quality of dose distribution is the top priority to maximize therapeutic efficacy and safety. Therefore, the SSO_20 with VDR is recommended for DCA-based SRS with the HDRS for BMs. In simultaneous treatment of multiple lesions, which was not examined in this study, the application of the SSO_20 will be essential since the difficulty of optimization definitely increases due to dose interference as the lesions become closer to each other.

In a typical DCA plan, the TV for dose prescription is the planning target volume (PTV) with adding an isotropic 1-2 mm margin to the GTV [4-11]. It is commonly optimized to cover the PTV boundary with a 70-80% IDS of the maximum or central dose as 100% [4-8,25,26]. The prescription dose is then assigned to the IDS covering a specific percentage of the PTV (e.g., *D*_95%_). However, the GTV marginal dose (e.g., *D*_V-0.01 cc_) is subject to considerable variability [23]. In a typical multi-arc setting, the MU allocation to each arc or beam weight is usually even, especially in forward planning [4-7]. However, the isotropic leaf margin settings, including negative values, have considerable limitations in achieving excellent dose conformity [12,13].

The inverse plan in this study was optimized to maximize the dose conformity of the prescription IDS to the GTV boundary and the steepness of the dose falloff outside the boundary, with the GTV itself as the TV. As a result, the GTV dose was highly heterogeneous, and the degree of dose inhomogeneity varied considerably. The allocation of MU values for each arc was automatically optimized and varied substantially, although the rotation range of the arcs was uniformly set at 120°. Furthermore, the SSO automatically optimized anisotropic leaf margin settings, including negative values, and eliminated the need for the mTV as a surrogate target [18,24]. The beam segments were likely designed to be smaller than the GTV contour while still conforming to the shape of the GTV to some extent, which is in contrast to typical three-dimensional conformal radiotherapy with fixed and sufficient leaf margins to the PTV boundary. The formation of segments smaller than the TV contour is an important factor in achieving the dose distribution suitable for SRS, leading to excellent dose conformity and steep dose falloff outside the TV boundary [5,6,26]. The SSO incorporates many of the optimization elements of VMA. In addition, the SSO enabled the dynamic shielding of some outermost leaves in the leaf movement direction with the diaphragms (jaws), which led to the practically <5 mm variable widths of the leaves [15-17]. The positions of the leaves and the diaphragms with the in-depth tracking are both controlled in increments of 0.1 mm.

Although the tCT was much longer than with the conventional pencil beam algorithm, the creation process of the dose distribution was automated, so the time required for the actual treatment plan, the time the planner spent operating the TPS, was significantly reduced. While the optimization is in progress, the planner can focus on other tasks.

The dose distribution design and dose regimen of this study were quite unique. The prescription dose was specified as the GTV *D*_V-0.01 cc_ so that the near-minimum biologically effective dose (BED) to the GTV would not decrease with increasing GTV [23]. Although priority was given to steepness of dose falloff outside the GTV boundary, it was intended to ensure an appropriate dose attenuation margin to cover microscopic brain invasion and inherent accuracy uncertainties [24,27]. In fact, the dose at 2 mm outside the GTV boundary was 70.7-94.0% relative to the prescription dose and was significantly higher with increasing GTV. Given the general tendency for the larger the GTV, the more significant the surrounding brain infiltration, the dose characteristics at 2 mm outside the GTV boundary were quite reasonable. However, even in the SSO_20, the dose prescription to the GTV *D*_V-0.01 cc_ showed substantially excessive PIV spillage outside the GTV. When applying a prescription dose equivalent to 80 Gy as a BED to the DCA, it may be safer to lower the GTV coverage, such as by assigning it as the *D*_V-0.035 cc_, with sufficient dose fractionation [27-30].

To determine whether the DCA should actually be adopted as an SRS method for BMs, a comparison of its merits with VMA with the HDRS is necessary [8,17,18,24]. These study results therefore warrant further investigation to directly compare DCA and VMA, including simultaneous irradiation with a single isocenter for multiple lesions [16,17]. In addition, it would be interesting to compare the DCA with VDR and the SSO_20 with other platforms, including a 2.5-mm leaf-width MLC and/or another TPS [7,8,11,17,26].

Limitations

Although the number of subjects (20) may be small, significant differences were observed in both the quality and efficiency, and the results would not change significantly even if the number of subjects were increased. It should be noted that the isotropic margin addition function differs depending on the TPS when assessing doses 2-4 mm outside and inside the GTV boundary [24]. There remain substantial opportunities for further improving the dose distributions by changing the arc rotation range and/or the collimator angle and by utilizing the biological or other physical cost functions in the optimization [24]. Regarding the setting of the value of the high-precision leaf positions, the results for other values, such as 10 (SSO_10) or 15 (SSO_15), are unknown. This study was limited to the analysis of the treatment planning stage and included no verification of the dose distributions by the point dose and volumetric measurements. In addition, the clinical usefulness of DCA with the HDRS needs to be proven in actual clinical outcomes.

## Conclusions

The inverse planning with VDR and the SSO_20 significantly improved the quality of DCA with the HDRS through anisotropic leaf adaptations, including minus leaf margins, and the <5 mm variable widths of the outermost leaves in the leaf movement direction by dynamic shielding with the diaphragms of the position controls in 0.1 mm increments. However, the SSO_20 with the VDR compromised the efficiency of planning and delivery with the longer planning time and the higher total MU per fraction. The results warrant further investigation to directly compare the DCA and the VMA for the SRS with the HDRS for single and multiple BMs.

## References

[1] Vogelbaum MA, Brown PD, Messersmith H (2022). Treatment for brain metastases: ASCO-SNO-ASTRO guideline. J Clin Oncol.

[2] Ladbury C, Pennock M, Yilmaz T (2023). Stereotactic radiosurgery in the management of brain metastases: a case-based Radiosurgery Society practice guideline. Adv Radiat Oncol.

[3] Pikis S, Protopapa M, Mantziaris G, Osama M, Sheehan J (2025). Stereotactic radiosurgery for brain metastases. Adv Cancer Res.

[4] Ohtakara K, Hayashi S, Hoshi H (2012). Characterisation of dose distribution in linear accelerator-based intracranial stereotactic radiosurgery with the dynamic conformal arc technique: consideration of the optimal method for dose prescription and evaluation. Br J Radiol.

[5] Ohtakara K, Hayashi S, Tanaka H, Hoshi H (2012). Consideration of optimal isodose surface selection for target coverage in micro-multileaf collimator-based stereotactic radiotherapy for large cystic brain metastases: comparison of 90%, 80% and 70% isodose surface-based planning. Br J Radiol.

[6] Tanyi JA, Doss EJ, Kato CM (2012). Dynamic conformal arc cranial stereotactic radiosurgery: implications of multileaf collimator margin on dose-volume metrics. Br J Radiol.

[7] Ohtakara K, Hayashi S, Tanaka H, Hoshi H (2011). Dosimetric comparison of 2.5 mm vs. 3.0 mm leaf width micro-multileaf collimator-based treatment systems for intracranial stereotactic radiosurgery using dynamic conformal arcs: implications for treatment planning. Jpn J Radiol.

[8] Torizuka D, Uto M, Takehana K, Mizowaki T (2021). Dosimetric comparison among dynamic conformal arc therapy, coplanar and non-coplanar volumetric modulated arc therapy for single brain metastasis. J Radiat Res.

[9] Mori Y, Kaneda N, Hagiwara M, Ishiguchi T (2016). Dosimetric study of automatic brain metastases planning in comparison with conventional multi-isocenter dynamic conformal arc therapy and Gamma Knife radiosurgery for multiple brain metastases. Cureus.

[10] Oshiro Y, Mizumoto M, Kato Y, Tsuchida Y, Tsuboi K, Sakae T, Sakurai H (2024). Single isocenter dynamic conformal arcs-based radiosurgery for brain metastases: dosimetric comparison with Cyberknife and clinical investigation. Tech Innov Patient Support Radiat Oncol.

[11] Oshiro Y, Kato Y, Mizumoto M, Sakurai H (2024). The impact of multileaf collimator size on single isocenter dynamic conformal arcs-based radiosurgery for brain metastases. Cureus.

[12] Ohtakara K, Nakabayashi K, Suzuki K (2023). Ten-fraction stereotactic radiosurgery with different gross tumor doses and inhomogeneities for brain metastasis of >10 cc: treatment responses suggesting suitable biological effective dose formula for single and 10 fractions. Cureus.

[13] Ohtakara K, Hoshi H (2014). Stereotactic radiotherapy planning using modified dynamic conformal arcs under considering the possibility for amended visual organ displacement resulting from early tumor shrinkage during treatment for perioptic involvement of myeloma. Int J Med Phys Clin Eng Radiat Oncol.

[14] Ogura K, Kosaka Y, Imagumbai T, Ueki K, Narukami R, Hattori T, Kokubo M (2017). Modifying the planning target volume to optimize the dose distribution in dynamic conformal arc therapy for large metastatic brain tumors. Jpn J Radiol.

[15] Saenz DL, Li Y, Rasmussen K, Stathakis S, Pappas E, Papanikolaou N (2018). Dosimetric and localization accuracy of Elekta high definition dynamic radiosurgery. Phys Med.

[16] Saenz D, Papanikolaou N, Zoros E (2021). Robustness of single-isocenter multiple-metastasis stereotactic radiosurgery end-to-end testing across institutions. J Radiosurg SBRT.

[17] Lai L, Li A, Liu J, Zhou L (2024). Comprehensively evaluating the performance of Elekta high-definition dynamic radiosurgery for hypofractionated stereotactic radiotherapy of multiple brain metastases. J Radiat Res Appl Sci.

[18] Thaper D, Kamal R, Singh G, Oinam AS, Yadav HP, Kumar R, Kumar V (2020). Dosimetric comparison of dynamic conformal arc integrated with segment shape optimization and variable dose rate versus volumetric modulated arc therapy for liver SBRT. Rep Pract Oncol Radiother.

[19] Lee S, Lee D, Verma V (2022). Dosimetric benefits of dynamic conformal arc therapy-combined with active breath-hold in lung stereotactic body radiotherapy. Med Dosim.

[20] Mesny E, Ayadi M, Dupuis P, Beldjoudi G, Tanguy R, Martel-Lafay I (2023). Clinical outcomes and lung toxicities after lung SABR using dynamic conformal arc therapy: a single-institution cohort study. Radiat Oncol.

[21] Iwai Y, Ozawa S, Ageishi T, Pellegrini R, Yoda K (2014). Feasibility of single-isocenter, multi-arc non-coplanar volumetric modulated arc therapy for multiple brain tumors using a linear accelerator with a 160-leaf multileaf collimator: a phantom study. J Radiat Res.

[22] Ohtakara K, Suzuki K (2024). Non-coplanar arc-involved beam arrangement with sufficient arc rotations is suitable for volumetric-modulated arc-based radiosurgery for single brain metastasis. Cureus.

[23] Ohtakara K, Suzuki K (2024). Proposal of an alternative near-minimum isodose surface DV-0.01 cc equally minimizing gross tumor volume below the relevant dose as the basis for dose prescription and evaluation of stereotactic radiosurgery for brain metastases. Cureus.

[24] Ohtakara K, Suzuki K (2024). Determining simple and effective cost functions for an efficient volumetric-modulated arcs-based stereotactic radiosurgery for single brain metastases using Monaco® planning system. Cureus.

[25] Walter YA, Dugas JP, Broekhoven BL, Jacobs TD, Han M, Wang CJ, Wu HT (2024). Effect of prescription isodose line on tissue sparing in linear accelerator-based stereotactic radiosurgery treating multiple brain metastases using dynamic conformal arcs. J Appl Clin Med Phys.

[26] Sagawa T, Ikawa T, Ohira S (2024). What is the optimal isodose line for stereotactic radiotherapy for single brain metastases using HyperArc?. J Appl Clin Med Phys.

[27] Ohguri T, Itamura H, Tani S, Shiba E, Yamamoto J (2025). High incidence of radiation-induced brain necrosis in the periventricular deep white matter: stereotactic radiotherapy for brain metastases using volumetric modulated arc therapy. Radiat Oncol.

[28] Matsuyama T, Kogo K, Oya N (2013). Clinical outcomes of biological effective dose-based fractionated stereotactic radiation therapy for metastatic brain tumors from non-small cell lung cancer. Int J Radiat Oncol Biol Phys.

[29] Loo M, Clavier JB, Attal Khalifa J, Moyal E, Khalifa J (2021). Dose-response effect and dose-toxicity in stereotactic radiotherapy for brain metastases: a review. Cancers (Basel).

[30] Seuntjens J, Lartigau EF, Cora S (2014). ICRU Report 91: prescribing, recording, and reporting of stereotactic treatments with small photon beams. J ICRU.

